# Curcumin Attenuates Oxidative Stress and Activation of Redox-Sensitive Kinases in High Fructose- and High-Fat-Fed Male Wistar Rats

**DOI:** 10.3797/scipharm.1408-16

**Published:** 2014-11-04

**Authors:** Nachimuthu Maithili Karpaga Selvi, Magadi Gopalakrishna Sridhar, Rathinam Palamalai Swaminathan, Ramalingam Sripradha

**Affiliations:** 1 Department of Biochemistry, Jawaharlal Institute of Postgraduate Medical Education and Research, Pondicherry – 605 006, India; 2 Department of Medicine, Jawaharlal Institute of Postgraduate Medical Education and Research, Pondicherry – 605 006, India

**Keywords:** Glutathione peroxidase, Catalase, Malondialdehyde, Total oxidant status, Oxidative stress

## Abstract

The present study was carried out to investigate the effects of curcumin on oxidative stress and redox-sensitive kinases in high fructose- and high-fat-fed rats. Sixty rats were randomly divided into six groups with ten animals each. Rats were fed with a standard rodent diet, high fructose diet (60%), and high-fat diet (30%). Curcumin was administered to control, high fructose and high fat diet groups for ten weeks. At the end of the study, body weight and blood glucose levels were measured. The antioxidant enzymes GSH (reduced glutathione), GPx (glutathione peroxidase), and catalase activities were estimated in the blood. MDA, TAS, and TOS were estimated in the plasma, liver, and kidney. Curcumin treatment decreased body weight and blood glucose levels in the rats fed with fructose and high-fat diet. Antioxidant enzymes and plasma TAS were significantly improved by curcumin treatment in high fructose-fed rats, whereas in high-fat-fed rats, there was an increase only in the GPx activity. Curcumin significantly attenuated the elevation of plasma MDA and TOS in both diet groups. Hepatic MDA and TOS were found to be decreased upon curcumin supplementation in both diet groups, whereas a decrease in the renal MDA levels was observed only in fructose-treated rats, not in fat-fed rats. Curcumin treatment elevated liver TAS in rats fed only with the fructose-rich diet. Curcumin showed a significant decrease in the oxidative stress index (OSI) in plasma, liver, and kidney tissues in both diet groups. ERK phosphorylation was significantly decreased in both diet groups by curcumin treatment. Similarly, curcumin reduced the phosphorylation of p38 MAPK only in the high fructose-fed rats, not in the high-fat-fed rats. No significant changes were found in JNK phosphorylation in both diet groups. Thus, curcumin may be effective in the management of diet-induced oxidative stress and could be explored as a therapeutic adjuvant against complications associated with obesity and diabetes.

## Introduction

Diabetes and obesity are major health problems worldwide. The prevalence of metabolic syndrome and its complications are being increasingly recognised. The intake of high amounts of fructose and fat is likely to lead to a constellation of abnormalities including insulin resistance, hypertriglyceridemia, heart disease, and obesity that mimic human metabolic syndrome [1, 2]. It is well documented that the dietary intake of fructose as well as a fat-rich diet causes enhanced production of free radicals and depletes the antioxidant levels, thereby creating a redox imbalance which ultimately results in oxidative stress [3, 4].

Oxidative stress is defined as the persistent imbalance between the production of reactive oxygen species (ROS) and antioxidant defense culminating in irreversible cellular alterations [5]. This redox imbalance is associated with various pathological conditions such as diabetes mellitus, obesity, and cardiovascular disease [6]. Recent evidence shows that the increased flux of FFA, glucose or hexosamine, and NADPH oxidase in diabetes leads to enhanced production of mitochondrial ROS resulting in oxidative damage [7]. ROS, such as superoxide anion (O_2_^−^), hydroxyl radical (OH^.^), and hydrogen peroxide (H_2_O_2_), which are produced during normal metabolic processes, are constantly buffered by endogenous antioxidants like reduced glutathione (GSH), superoxide dismutase (SOD), glutathione peroxidase (GPx), and catalase [5, 8]. Overproduction of ROS or a reduced level of antioxidants, or both, lead to oxidative damage of membrane proteins, lipids, and DNA.

Imbalance in the antioxidant system also leads to excess production of ROS resulting in the activation of stress-sensitive signaling pathways called mitogen activated protein kinases (MAPK) [9]. Members of MAPK, such as extracellular signal-regulated kinases (ERK), c-Jun NH2-terminal kinases (JNK), and p38 kinases, are MAPK cascades activated by cytokines, hormones, and various cellular stressors such as oxidative stress and endoplasmic reticulum stress [9, 10]. As a consequences of these, the formation of gene products, which cause cellular damage, are ultimately responsible for complications of metabolic diseases. The modulation of redox imbalance by treatment with antioxidants can significantly alter oxidative stress resistance and the accumulation of oxidative damage.

In recent years, the use of alternative therapeutic approaches has been explored. Many known plants contain phytochemicals, some of which are polyphenolic compounds which exhibit potent antioxidant activity and can be used to alleviate the complications associated with metabolic syndrome. Hence, the use of dietary phytochemicals which attenuate the activation of these stress-sensitive signaling pathways and enhance the endogenous antioxidant defensive mechanism are being considered as dietary adjuvants. Curcumin (diferuloylmethane) is the active component derived from *Curcuma longa*. Curcumin is a potent scavenger of a variety of reactive oxygen species including superoxide anion radicals, hydroxyl radicals [11], and nitrogen dioxide radicals [12], and these protective effects are attributed to its antioxidant property. Studies have also shown that curcumin exhibits strong antioxidant activity and plays a vital role against oxidative stress-mediated diseases like diabetes, obesity, cardiovascular disease, etc. [13]. However, the molecular mechanism by which curcumin decreases the oxidative stress remains unclear. Hence, the present study was carried out to investigate the effect of curcumin on oxidative stress and redox-sensitive kinases in high fructose- and high-fat-fed rats.

## Materials and Methods

### Chemicals

All the chemicals used for the various assays were of molecular reagent grade and were obtained from Sigma Aldrich (USA), Merck (India), SRL (India). The primary antibodies ERK ½, Phosho ERK ½, p38, and Phospho p38 were purchased from Cell Signaling Technology, Inc. (Danvers, MA, USA). JNK and phospho-JNK were obtained from Pierce (Thermo Scientific, USA). The peroxidase-conjugated secondary antibodies were from Santa Cruz (Santa Cruz Biotechnology, Santa Cruz, CA, USA). The nitrocellulose membrane and CL–Xposure films were from Amersham (Amersham Hybond-ECL membrane, GE Healthcare, Little Chalfont, Buckinghamshire, UK). The enhanced chemiluminescence substrate (ECL) was from Pierce, West Pico Super Signal (Thermo Fisher Scientific, Marietta, USA).

### Animals and Treatment

Five-month-old male Wistar rats of body weight ranging from 250–300 g were used for this study. All experimental procedures were approved by the Institutional Animal Ethics Committee. They were allowed access to water and food ad libitum. All of them received standard pellet diet for one week. After acclimatization, the rats were randomly divided into six experimental groups with 10 rats in each group. The experiment was carried out for 10 weeks. Curcumin (200 mg/kg body weight) was prepared in 0.1% carboxymethylcellulose and administered by oral gavage [14].

Group 1: Control rats were fed with standard rodent chow.
Group 2: Control + curcumin group received standard rodent chow and curcumin for 10 weeks.
Group 3: High fructose (HF) group was fed with 60% fructose mixed with standard rodent chow.
Group 4: HF + curcumin group was administered with curcumin and HF for 10 weeks.
Group 5: High-fat diet (HFD) rats were fed with high-fat diet mixture.
Group 6: High-fat diet (HFD) + curcumin group was administered with curcumin and HFD for 10 weeks.

The control rats received the standard pellet, and the energy of the control diet was 3.2 kcal/g. The fructose diet contained 60% fructose (w/w), 11% fat, 29% protein [15] and was prepared by mixing 60% of fructose with the standard rodent chow. The fructose diet provided 60% of the total calories. The non-purified high-fat diet was prepared as described [16] with 59% of total calories derived from fat, 21% from protein, and 20% from carbohydrate. The energy content of the high-fat diet was 5.2 kcal/g.

### Composition of the High-Fat Diet


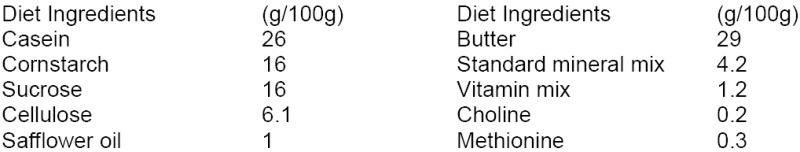


At the end of the experiment, fasting blood samples were collected. The antioxidant parameters like whole blood reduced glutathione, plasma total antioxidant status, erythrocyte glutathione peroxidase, and catalase activity were estimated. MDA and TOS were estimated in the plasma. After 10 weeks of the experimental period, the animals were sacrificed under anesthesia. Liver and kidney tissue mass were frozen immediately in liquid nitrogen and stored at −80°C for subsequent analysis. The total oxidant status and stress-sensitive signaling pathways were studied in the liver and kidney.

### Estimation of Oxidant and Antioxidant Status in Plasma, Liver, and Kidney

Liver and kidney homogenates were prepared using 0.1 M ice-cold Tris-HCI buffer (pH 7.5, 10% W/V). The homogenates were then centrifuged at 14,000 × *g* for 15 min at 4°C. The supernatants were used for the estimation of oxidant and total antioxidant status. Malondialdehyde levels were estimated according to the method of Okhawa *et al*. [17]. The protein content in the liver and kidney homogenates were measured by the method of Lowry *et al*. [18]. Plasma glucose was measured by the glucose oxidase-peroxidase (GOD-POD) method using standard reagent kits adapted to clinical chemistry Analyser [Olympus AU 400 (Siemens, Japan)]. The total antioxidant status in plasma and tissue samples was analysed by the FRAP method [19]. The total oxidant status in plasma and tissue samples was estimated by Ozcan Erel *et al*. [20]. The whole blood glutathione content was measured by the method of Buetler *et al*. [21]. Catalase enzyme activity in erythrocytes was estimated by the method of Aebi *et al*. [22]. The plasma MDA level was estimated using HPLC (Shimadzu, Japan) [23]. The glutathione peroxidase activity in erythrocytes was determined by the method of Wendel *et al*. [24].

### Immunoblot Analyses of Stress Signaling in the Liver

Liver homogenates were prepared in a lysis buffer (50 mM Tris, pH 8.0, 1% Nonidet P-40, 0.25% sodium deoxycholate, 150 mM sodium chloride, 0.1% sodium dodecyl sulphate (SDS), 1 mM sodium fluoride, 1 mM sodium orthovanadate, 1 mM phenylmethylsulfonyl fluoride, 20 mM dithiothreitol, 1 mM aprotinin, and 0.5% okadaic acid). After homogenization, the samples were centrifuged at 10,000 g for 30 min and the protein contents were estimated by Lowry’s method and the proteins were resolved by 12% sodium dodecyl sulphate polyacrylamide gel electrophoresis (Mini Protean II System, Bio-Rad). The resolved proteins were transferred onto a nitrocellulose membrane (Sigma, USA) and blocked with 5% BSA or 5% nonfat dry milk. The membranes were immunoblotted with antibodies specific to phospho-ERK ½, phospho-p38, and phospho-JNK followed by incubation with horseradish peroxidase conjugated anti-rabbit IgG (1:5000 dilutions) or anti-mouse IgG for 1 h at room temperature. Membranes were stripped of all bound antibodies and then reprobed with antibodies specific to ERK ½, p38, and JNK. Band intensities were visualized by the enhanced chemiluminescence method using an ECL kit (Pierce, Thermo Scientific Inc, USA). Images were captured with a GS-800 densitometer and quantified using Quantity One Software (Biorad Laboratories Inc., USA).

### Statistical Analysis

Results were expressed as mean ± SD. The analysis was done by one-way repeated measurements of analysis of variance (ANOVA) followed by an appropriate post hoc test using the Statistical Package of Social Service (SPSS, Version 19.0). A p-value less than 0.05 was considered as statistically significant.

## Results

### Effect of Curcumin on Body Weight and Blood Glucose Levels

Both high fructose and high-fat diet feeding significantly increased body weight when compared with the control group. Upon curcumin supplementation, rats fed the high fructose and high-fat diet reduced body weight gain 9.3% and 8.5%, respectively, when compared with the high fructose and high-fat-fed groups. Plasma glucose levels were elevated in both diet groups and the addition of curcumin to both diets reduced the increase by 18% and 16%, respectively, when compared with high fructose and high-fat-fed groups.

**Fig. 1:**
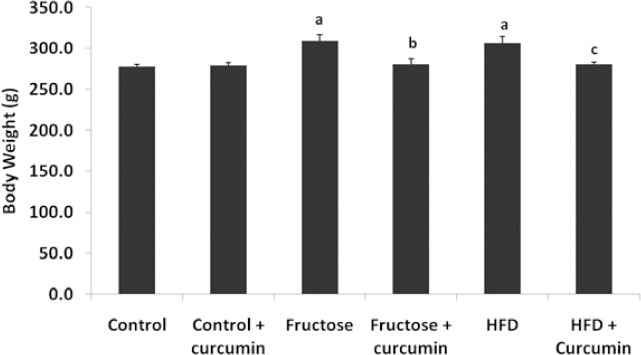
Effect of curcumin on body weight at the end of the 10^th^ week. Data were expressed as mean ± SD. (n=10, P<0.05, ^a^ in comparison with control, ^b^ in comparison with fructose group, ^c^ in comparison with HFD). Differences between the groups were analysed using one-way ANOVA with the Tukey post hoc method. P< 0.05 is considered statistically significant. HFD= High-fat diet.

**Fig. 2:**
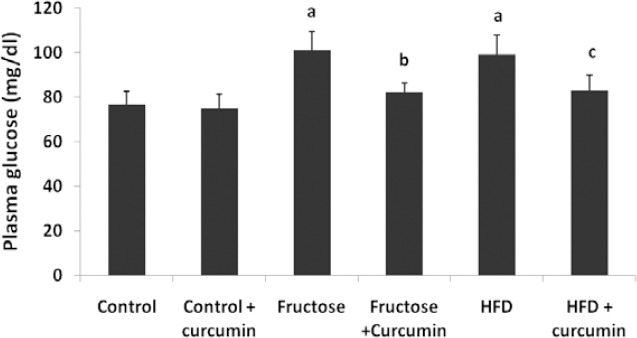
Effect of curcumin on plasma glucose levels. Data were expressed as mean ± SD. (n=10, P<0.05, ^a^ in comparison with control, ^b^ in comparison with fructose group, ^c^ in comparison with HFD). HFD= High-fat diet. Differences between the groups were analysed using one-way ANOVA with the Tukey post hoc method.

### Effect of Curcumin on Blood Antioxidant Enzyme Activities

GSH, GPx, and catalase activities were significantly decreased in fructose-fed and high-fat-fed rats (Table 1), whereas the plasma TAS was significantly reduced in high fructose-fed rats, but not in high–fat-fed rats. Administration of curcumin along with the fructose diet significantly increased the TAS 57% (P < 0.05) and GSH levels 58% (P < 0.05), GPx 56% (P < 0.001), and catalase activities 34% (P < 0.05) in comparison to the fructose group. These effects were not observed when the rats were fed with a high-fat diet, except for GPx activity which was found to be increased about 32% (P < 0.05) with curcumin treatment.

**Tab. 1:**
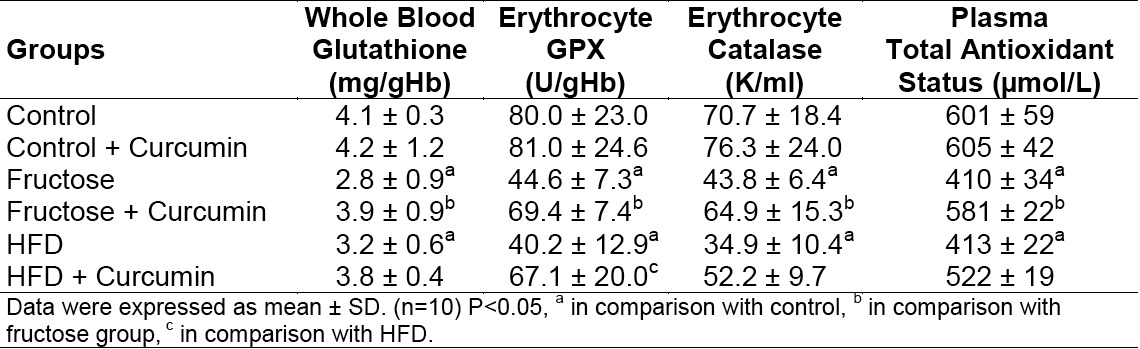
Effect of curcumin on blood antioxidant parameters (GSH, GPx, Catalase, and TAS) in high fructose-fed and high-fat fed rats

### Effect of Curcumin on Plasma Oxidative Stress

Both high-fat feeding as well as high fructose feeding in rats led to increased plasma oxidative stress parameters: MDA, TOS, and OSI (Table 2). The percentage change in the OSI of blood was 73.3% (P < 0.001) and 63.5% (P < 0.001), respectively, in high fructose- and high-fat-fed rats. OSI indicates the severity of oxidative stress. Treatment with curcumin reduced MDA, TOS, and OSI about 63% (P < 0.001), 45% (P < 0.001), and 59% (P < 0.001), respectively, in high fructose-fed rats and 53% (P < 0.001), 25% (P < 0.05), and 36% (P < 0.001), respectively, in high-fat-fed rats. The severity of oxidative stress was more pronounced in high fructose- than high-fat-fed rats.

**Tab. 2:**
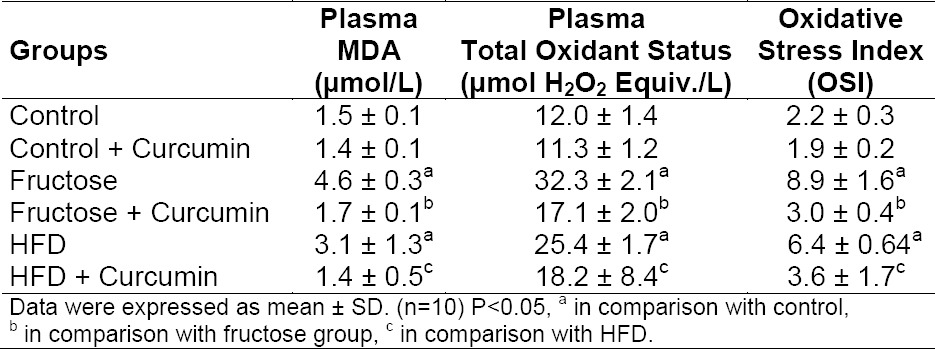
Effect of curcumin on plasma oxidative stress markers in high fructose- and high-fat-fed rats

### Effect of Curcumin on Hepatic and Renal Oxidative Stress Markers

Both high fructose- and high-fat-fed rats displayed significant increase in MDA level, TOS status, and reduced TAS in the liver when compared with the control group (Table 3). The percentage change in hepatic OSI was 65% (P < 0.001) and 53% (P < 0.001), respectively, in high fructose- and high-fat-fed rats when compared to the control group.

**Tab. 3:**
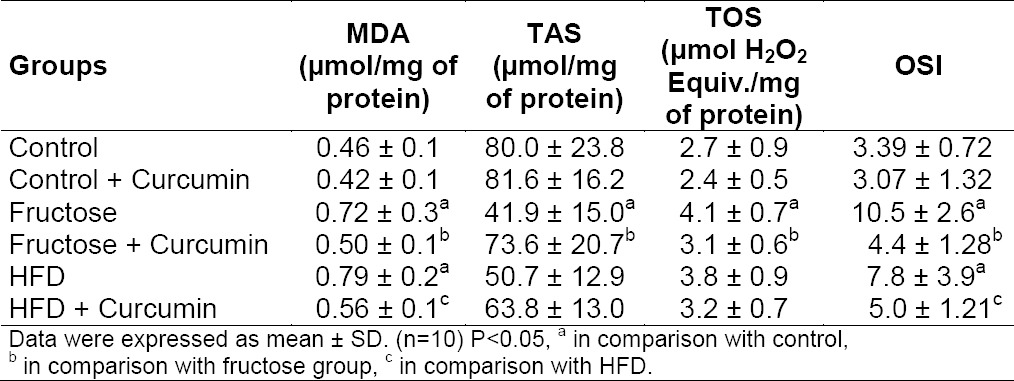
Effect of curcumin on oxidative stress markers in liver of high fructose- and high-fat-fed rats

Curcumin administration in high fructose-fed rats decreased hepatic TOS status (P < 0.05), MDA (P < 0.05) levels, and improved total antioxidant status (P < 0.001). The OSI levels of high fructose-fed and high-fat-fed rats were 56% and 31% lower, respectively, after curcumin treatment. In the high-fat-fed group, only the MDA level improved and no significant changes were found in the rest of the parameters.

It was found that MDA levels were raised in kidneys on high fructose and high-fat treatment (Table 4). Curcumin, when supplemented with the same diet, significantly decreased the renal MDA formation in high fructose-fed rats. The percentage decrease in OSI of the kidneys was 13% and 16%, respectively, in high fructose and high-fat treatment when compared to the control group.

**Tab. 4:**
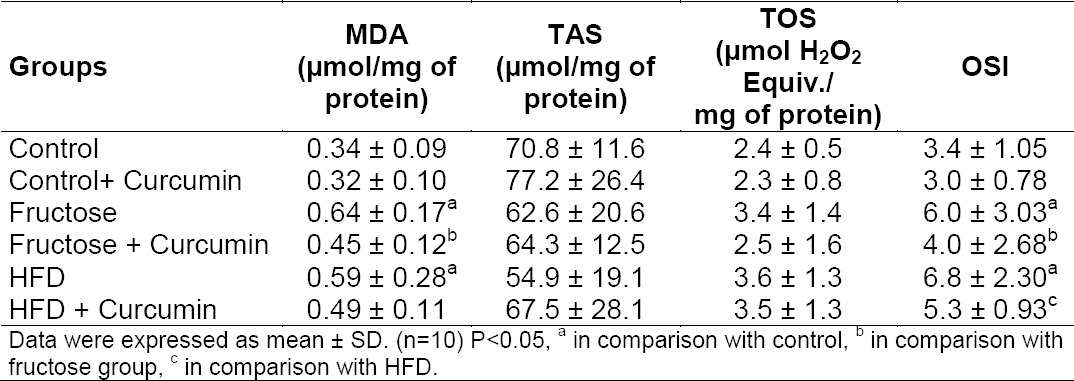
Effect of curcumin on oxidative stress markers in kidney of high fructose- and high-fat-fed rats

### Effect of High Fructose Diet, High-Fat Diet, and Curcumin Supplementation on Stress-Sensitive Kinases

**Fig. 3:**
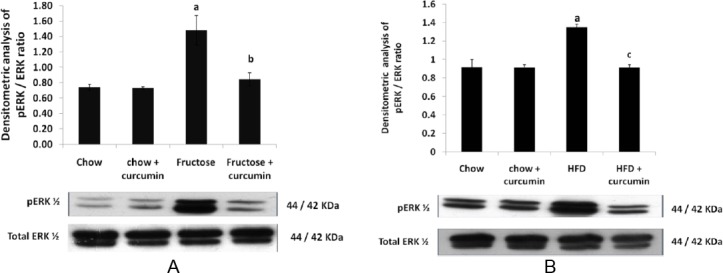
Western blot analysis of the effect of curcumin on ERK ½ phosphorylation levels. (A) ERK ½ phosphorylation levels in the high fructose diet. (B) ERK ½ phosphorylation levels in the high-fat diet. Data were analyzed by one-way ANOVA. Data were expressed as mean ± SD. (n=3) P<0.05, ^a^ in comparison with control, ^b^ in comparison with fructose group, ^c^ in comparison with HFD.

**Fig. 4:**
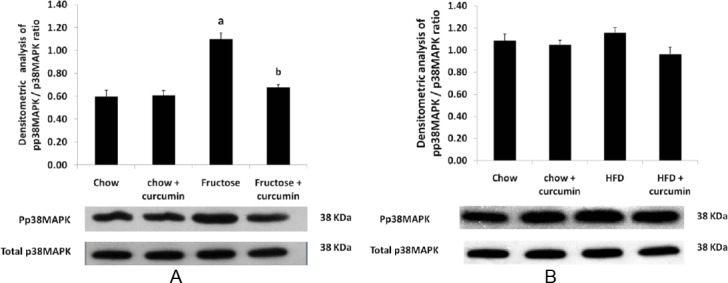
Western blot analysis of the effect of curcumin on p38 phosphorylation levels. (A) p38 phosphorylation levels in the high fructose diet. (B) p38 phosphorylation levels in the high-fat diet. Data were analyzed by one-way ANOVA. Data were expressed as mean ± SD. (n=10) P<0.05, ^a^ in comparison with control, ^b^ in comparison with fructose group, ^c^ in comparison with HFD

**Fig. 5:**
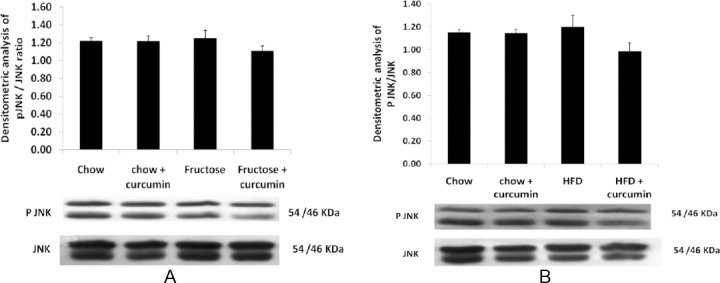
Western blot analysis of the effect of curcumin on JNK phosphorylation levels. (A) JNK phosphorylation levels in the high fructose diet. (B) JNK phosphorylation levels in the high fat diet

The high fructose and high-fat diet group significantly increased ERK½ phosphorylation in comparison to the control group (Figures 3A & B). P38 phosphorylation was also elevated in the high fructose diet when compared to the control group (Figure 4 A & B). Treatment with curcumin in the high fructose and high-fat diet group markedly inhibited the ERK and p38 pathway activation when compared to the high fructose and high-fat diet group, whereas no significant change was found in JNK phosphorylation in both groups (Figures 5A & B).

## Discussion

Oxidative stress is considered as one of the risk factors which contribute to the onset of insulin resistance and its complications, type 2 diabetes and obesity [25]. It has been proposed that the consumption of dietary fructose and fat-rich products increases the prevalence of oxidative stress associated with insulin resistance. Curcumin (diferuloylmethane) is a phytochemical, the active component in the turmeric rhizome, which is a widely used spice and a therapeutic agent for various ailments in the traditional medicine of Southeast Asian countries. Many studies have demonstrated the antioxidant properties of curcumin both *in vivo and in vitro* [26].

Available evidence indicates that hyperglycemia and free fatty acids (FFA) in diabetes and obesity result in the generation of reactive oxygen species (ROS), ultimately leading to increased oxidative stress. Hyperglycemia-induced oxidative stress may be due to the increased production of mitochondrial ROS, non-enzymatic glycation of proteins [27], and glucose auto-oxidation. Enhanced FFA also increase mitochondrial uncoupling and β-oxidation, thereby leading to the increased generation of ROS resulting in oxidative stress [28]. The activation of stress-sensitive signaling pathways is a major consequence of oxidative stress [29]. Thus, treatment aimed at reducing the severity of oxidative stress and activation of stress-sensitive signaling pathways could be used as therapeutic strategies in the management of complications induced by oxidative stress in diabesity.

In our study, curcumin treatment decreased body weight and blood glucose levels in rats fed with a high fructose diet and a fat-rich diet at the end of the study. This result was consistent with previous findings. It has been reported that curcumin lowers TG and FFA levels in high-fat-fed hamsters [30] and this hypolipidemic property may be the reason for the reduction in body weight. As shown in previous studies, curcumin has a beneficial role in improving insulin resistance and lowering blood glucose in db/db mice through the elevation of plasma insulin levels, which enhances the activation of glycolysis and inhibits gluconeogenesis [31].

In the present study, we found a significant increase in plasma MDA and TOS, with an attendant decrease in plasma TAS, whole blood GSH, and antioxidant enzymes GPx and catalase in both high-fat-fed rats and high fructose-fed rats. Fructose feeding induces free radical formation by downregulation of the HMP shunt pathway that generates a reduced environment in the form of NADPH and NADH [32]. Impairment in the generation of NADH results in ROS production. Furthermore, it has been postulated that fructose can accelerate free radical production similar to glucose. Reactive oxygen species (ROS) can themselves reduce the activity of antioxidant enzymes such as catalase and GPx [33]. Previous studies have shown that increased ROS production from accumulated fat leads to increased oxidative stress in blood, affecting other organs including the liver, skeletal muscles, and aorta [34].

A decrease in the antioxidant status exacerbates the production of reactive oxygen species O_2_^−^ and H_2_O_2_, which in turn causes peroxidation of membrane lipids leading to the formation of lipid byproducts, MDA etc, thereby increasing the plasma MDA levels, TOS and OSI [8]. The TAS/TOS (OSI) ratio was increased in both diet groups. The OSI index indicates the degree of oxidative stress and reflects the present oxidative stress. The percentage change in OSI was 73.3% and 63.5% in fructose-fed and high-fat-fed rats, respectively, when compared to the control group. The severity of redox imbalance is greater in the fructose-fed group than the high-fat-fed group. Here we have shown that both high-fat feeding as well as high fructose-feeding in rats led to oxidative stress and the severity of oxidative stress was found to be more pronounced in the high fructose-fed rats than in the rats fed with a high-fat diet as indicated by OSI. Curcumin treatment decreased the OSI of the plasma in both diet groups.

Treatment with curcumin restored erythrocyte GPx, catalase activities, and plasma TAS in the fructose-fed rats and GPx activity in the high-fat-fed rats. It also reversed the depletion of erythrocyte GSH in fructose-fed rats, thereby augmenting the antioxidant defense system. It also inhibited the lipid peroxidation product, MDA, and TOS/TAS ratio. Our results were consistent with previous reports where curcumin reversed the diminished erythrocyte Gpx activity and attenuated the increase of lipid peroxidation product (MDA). The endogenous antioxidant, GPx is more effective than SOD and catalase in scavenging free radicals [35]. It has already been reported that the free radical scavenging mechanism is one of the major antioxidant defense systems against ROS-induced metabolic changes [36]. Hence, both antioxidant enzymes (GPx, catalase) appear to play an important role in scavenging H_2_O_2_ generated by the action of SOD, thereby protecting the cells from various oxidative damage. Curcumin exerts its protective effect by modulating lipid peroxidation and augmenting the antioxidant defense system, and its effects are attributed to the presence of the hydroxyl groups and methylene group of the β-diketone moiety [37]. Previous reports have shown significant reduction of MDA and increased SOD and catalase activity in diabetic rats upon curcumin treatment [38].

Both high fructose- and high-fat-fed rats showed a significant increase in MDA levels, TOS, OSI, and reduced TAS in the liver when compared with the control group. In the kidney, a significant elevation of MDA was noted in both diet groups. The percentage change in hepatic OSI was 65% and 53%, respectively, in the fructose- and high-fat-fed rats when compared to the control groups. These hepatic changes further support the concept that the severity of fructose-mediated redox imbalance is greater than high-fat diet-induced oxidative stress. The above-mentioned changes were markedly reversed by treatment with curcumin and renal MDA of fructose-fed rats was brought back to normal. These results were in agreement with the previous study. Studies have shown that curcumin significantly enhanced the synthesis of antioxidant enzymes such as SOD, CAT, and GPx in rat livers and attenuated the increase of lipid peroxidation [11, 39].

ERK ½, JNK, and p38 can be activated by a variety of exogenous and endogenous stress-inducing stimuli including hyperglycemia, ROS, oxidative stress, osmotic stress, and pro-inflammatory cytokines. Chronic activation of the MAPK pathway is often associated with various pathological conditions like diabetes, obesity, ischemia/reperfusion injury, infectious disease, and neuronal disease [40]. In the present study, we have shown that ERK ½ and p38 phosphorylation were increased 1.6-fold and 1.3–fold, respectively, in the fructose-fed group when compared to the control group. In the high-fat-fed group, there was a two-fold increase in the ERK phosphorylation, while no significant change was observed in the phosphorylation of p38. Both diet groups did not show any significant change in JNK phosphorylation. This finding is consistent with previous studies where increased basal levels of phosphorylated ERK ½ and p38 MAPK were observed in adipocytes [41] or skeletal muscle [42] of obese, insulin resistant, and type 2 diabetic subjects.

Activation of the p38 MAPK pathway occurs in response to hyperglycemia in diabetes and obesity, which causes cellular damage and is ultimately responsible for their long-term complications [43]. Activation of stress-sensitive kinases like p38 and ERK ½ stimulates the serine phosphorylation of 1RS-1/IRS-2 which reduces insulin-stimulated IRS-1 tyrosine phosphorylation and insulin sensitivity which triggers the early onset of diabetes [44]. Intervention and therapy that alters or disrupts these mechanisms may serve to reduce the risk of insulin resistance and the development of complications in diabetes.

Our study found that curcumin supplementation along with a high fructose and high-fat diet inhibits the phosphorylation of ERK and p38. Curcumin decreases the phosphorylation of the stress-sensitive kinases by inhibiting oxidative stress and augmenting the antioxidant defense system. Thus, the antioxidant property of curcumin helps in scavenging free radicals generated in various conditions and halts the activation of stress-sensitive kinases, thereby preventing the cell damage and its associated metabolic derangements.

## Conclusion

Dietary intake of either high fructose or high-fat leads to the development of oxidative stress. Further, the severity of oxidative stress is likely to be higher when a fructose-rich diet is consumed than when a fat-rich diet is taken. Curcumin administration improved redox balance by reducing oxidants status and enhancing antioxidant defense systems. Thus, the protective role of curcumin against diet-induced oxidative stress may be useful in developing appropriate adjuvant strategies in the treatment of oxidative stress-mediated complications seen in the early stages of obesity and type 2 diabetes mellitus.
